# Microbial virulence, molecular epidemiology and pathogenic factors of fluoroquinolone-resistant *Haemophilus influenzae* infections in Guangzhou, China

**DOI:** 10.1186/s12941-018-0290-9

**Published:** 2018-11-23

**Authors:** Dingqiang Chen, Shuxian Wen, Donghua Feng, Ruirui Xu, Junyan Liu, Brian M. Peters, Danhong Su, Yongping Lin, Ling Yang, Zhenbo Xu, Mark E. Shirtliff

**Affiliations:** 1 Department of Laboratory Medicine, The First Affiliated Hospital of Guangzhou Medical University, Guangzhou Medical University, Guangzhou, 510120 China; 2 School of Food Science and Engineering, South China University of Technology, Guangzhou, 510640 China; 3 Department of Clinical Pharmacy, College of Pharmacy, University of Tennessee Health Science Center, Memphis, TN 38163 USA; 4 Guangdong Province Key Laboratory for Green Processing of Natural Products and Product Safety, Guangzhou, 510640 China; 5 Department of Microbial Pathogenesis, School of Dentistry, University of Maryland, Baltimore, MD 21201 USA; 6 Department of Laboratory Medicine, Zhujiang Hospital, Southern Medical University, Guangzhou, 510280 China

**Keywords:** *Haemophilus influenzae*, Fluoroquinolones, Quinolone resistance-determining regions, ST1719

## Abstract

**Background:**

Fluoroquinolone-resistant *Haemophilus influenzae* (FRHI) has been reported worldwide but remain unclear in China.

**Methods:**

A total of 402 *H. influenzae* isolates collected from 2016 to 2017 were included. Antimicrobial susceptibility on 10 antibiotics was performed, and minimum inhibitory concentration of ciprofloxacin- and nalidixic acid-resistant strains were further determined by E-test strips, with risk factors also evaluated. Strains with resistance or reduced susceptibility to ciprofloxacin were subjected to sequencing of the quinolone resistance-determining regions (QRDR) and plasmid-mediated quinolone resistance genes by sequencing, with multi-locus sequence typing.

**Results:**

2.2% of *H. influenzae* strains were non-susceptible (7/402, 1.7%) or susceptible (2/402, 0.5%) to ciprofloxacin but NAL-resistant by E-test, and multidrug resistance was more common in fluoroquinolones non-susceptible *H. influenzae* group (*p* = 0.000). Infection risk factors included invasive procedure (*p *= 0.011), catching cold/previous contact with someone who had a cold (*p *= 0.019), fluoroquinolones use during previous 3 months (*p *= 0.003). With none of mutations obtained in *gyrB*, *parE* and other plasmid-mediated quinolone resistance genes, 7 and 4 strains were found for Ser-84-Leu substitutions in *gyrA* and one amino acid substitution in the QRDR of *gyrA* linked with one amino acid substitution in the QRDR of *parC*, respectively. In addition, five sequence types (ST) were identified, with ST1719 firstly found.

**Conclusions:**

For the first time, this study has reported the incidence, risk factors, molecular determinants on fluoroquinolones resistance and ST of FRHI strains in mainland China, representing the first evidence of mutation of gyrA and parC in China and the new ST1719 worldwide.

## Background

*Haemophilus* influenzae is a conditional pathogen that commonly colonizes in the nasopharynx of human beings. As a leading cause for community-acquired pneumonia (CAP), *H. influenza* is also a major cause of acute otitis media (AOM) and bronchitis in young children [1], responsible for sever meningitis and septicemia responsible for 371,000 deaths per year globally [2].

Fluoroquinolones (FQs), a frequently used antibiotic for *H. influenzae* infections, was recommended for elderly patients or patients with underlying diseases according to the guidelines for CAP in Japan [3]. After the first report of FQs-resistant *H. influenzae* (FRHI) in 1993, this pathogen has spread worldwide and increased rapidly [4, 5]. Though a number of previous studies on FRHI from Hong Kong and Taiwan were available, the incidence of FRHI was controversial [4, 6–10]. In Taiwan, resistance rate of fluoroquinolone has increased rapidly over recent years [4, 7], and levofloxacin-resistance *H. influenzae* has been found to spread in the nursing home residents [7, 11] and was related to regional predominance of certain STs [4, 12]. In Hong Kong, the carriage rate of *H. influenzae* was 28.5% in children aged 2–6 years and this was the first report about resistance to quinolones among *H. influenzae* isolates in children [6]. However, despite a regional cross-sectional study recently from Shanghai which reported an 8% *H. influenzae* carriage in healthy children (aged 12–18 months) and sensitivity to all tested antimicrobials except 12.2% ampicillin resistance to ampicillin (with none of FRHI found) [10], surveillance on FRHI still remains significantly limited in mainland China.

Although resistance to FQs in *H. influenzae* remains low, levofloxacin treatment failure in *H. influenzae* pneumonia has been reported [13]. A patient with *H. influenzae* pneumonia died after several days of systemic treatment with levofloxacin and several mutations in the quinolone resistance-determining regions (QRDR) of DNA gyrase and topoisomerase IV genes of the *H. influenzae* strain were detected [14]. Resistance to FQs is due mainly to chromosome-mediated mutations in the QRDRs of the genes encoding DNA gyrase and topoisomerase IV [15], which is including *gyrA*, *gyrB*, *parC* and *parE*. In general, resistance to FQs developed in a stepwise manner. Mutation in DNA gyrase *gyrA*, which was regarded as the first-step mutants, can even occur in *H. influenzae* that FQs MICs remains in the susceptible range according to the current breakpoints in CLSI [6]. Carmen et al. believe that FQ resistance is acquired gradually with increasing numbers of mutations [16]. Nalidixic acid can be used in tests to screen for the detection of decreased susceptibility to quinolones in *H. influenzae* [6, 17, 18], it can find out isolates with QRDR mutant while quinolones MICs were still susceptible according to the breakpoint in CLSI.

In addition to the controversial FRHI incidence in Hong Kong and Taiwan and unavailability on FRHI study in mainland China [6, 7], antimicrobial resistance mechanism and sequence types (ST) of FRHI in mainland China remains unclear. In Guangzhou representative of Southern China, a number of longitudinal surveillances had been previously performed on various bacteria [18–21], including antimicrobial resistance and molecular mechanisms. Conducted in the same location, this study aimed to investigate the molecular epidemiology and risk factors of FRHI infections.

## Methods

### Species identification

The study was carried out at The First Affiliated Hospital of Guangzhou Medical University, a comprehensive teaching hospital located in Guangzhou representative of Southern China and featured by the leading respiratory diseases study and treatment. *H. influenzae* isolates were recruited from patients during January 2016 to January 2017. The samples were grown on chocolate agars and incubated for 24 h at 37 °C in air with 5% CO_2_. Suspicious colonies were removed from the surface of the plate for species identification, meanwhile, colonies (two to three) were homogenized in a reaction tube containing nanopure water (100 μl) [22]. Species identification were performed by matrix-assisted laser desorption ionization time-of-flight mass spectrometry (MALDI-TOF MS, AB biomerieux), and confirmed by 16S rRNA sequencing [23] (Primers were list in Table 1). DNA extracts were performed by boiling the bacterial suspensions [22]. The bacterial suspensions were heated to 100 °C in a boiling water bath for 10 min and centrifuged at 10,000×*g* for 10 min, then DNA was used as template and amplified with the following polymerase chain reactions (PCR) procedure: denaturation at 95 °C for 5 min, followed by 30 cycles of 95 °C for 30 s, 55 °C for 30 s, and 72 °C for 90 s. The samples were then extended at 72 °C for a further 10 min, hold to 4 °C. The sequences of the PCR products were compared with known 16S rRNA gene sequences in the GenBank (http://www.ncbi.nlm.nih.gov) as described previously [23, 24].

**Table 1:** Primers used in this study

Genes	Primers	Sequences	References
*16S rRNA*	*LPW55*	AGTTTGATCCTGGCTCAG	[19]
	*LPW56*	AGGCCCGGGAACGTATTCAC	
*gyrA*	*gyrA*-F	CCGCCGCGTACTGTTCT	[21]
	*gyrA*-R	CCATTTGCTAAAAGTGC	
*gyrB*	*gyrB*-F	GGAAAATCCTGCAGATGC	[21]
	*gyrB*-R	AAGCAACGTACGGATGTG	
*parC*	*parC*-F	TGGTTTAAAACCCGTTCA	[21]
	*parC*-R	AGCAGGTAAATATTGTGG	
*parE*	*parE*-F	GAACGCTTATCATCACGCCA	[21]
	*parE*-R	AGCATCCGCGAGAATACAGA	
*qnrA*	*QnrA*-F	AGAGGATTTCTCACGCCAGG	[22]
	*QnrA*-R	TGCCAGGCACAGATCTTGAC	
*qnrB*	*QnrB*-F	GGMATHGAAATTCGCCACTG	[22]
	*QnrB*-R	TTTGCYGYYCGCCAGTCGAA	
*qnrS*	*QnrS*-F	GCAAGTTCATTGAACAGGGT	[22]
	*QnrS*-R	TCTAAACCGTCGAGTTCGGCG	
*Aac(6′)-Ib-cr*	*aac (6′)-Ib-cr*-F	TTGCGATGCTCTATGAGTGGCTA	[22]
	*aac (6′)-Ib-cr*-R	CTCGAATGCCTGGCGTGTTT	
*qepA*	*qepA*-F	AACTGCTTGAGCCCGTAGAT	[22]
	*qepA*-R	GTCTACGCCATGGACCTCAC	
*oqxA*	*oqxA*-F	AGTCCATACCAACCTCGTCTCC	[22]
	*oqxA*-R	GCGTGGCTTTGAACTCTGC	
*oqxB*	*oqxB*-F	CCACCCTTAACTGATCCCTAA	[22]
	*oqxB*-R	CGCCAGCTCATCCTTCAC	
*adk*	*adk*-F	GGTGCACCGGGTGCAGGTAA	[23]
	*adk*-R	CCTAAGATTTTATCTAACTC	
*atpG*	*atpG*-F	ATGGCAGGTGCAAAAGAGAT	[23]
	*atpG*-R	TTGTACAACAGGCTTTTGCG	
*frdB*	*frdB*-F	CTTATCGTTGGTCTTGCCGT	[23]
	*frdB*-R	TTGGCACTTTCCACTTTTCC	
*fucK*	*fucK*-F	ACCACTTTCGGCGTGGATGG	[23]
	*fucK*-R	AAGATTTCCCAGGTGCCAGA	
*mdh*	*mdh*-F	TCATTGTATGATATTGCCCC	[23]
	*mdh*-R	ACTTCTGTACCTGCATTTTG	
*pgi*	*pgi*-F	GGTGAAAAAATCAATCGTAC	[23]
	*pgi*-R	ATTGAAAGACCAATAGCTGA	
*recA*	*recA*-F	ATGGCAACTCAAGAAGAAAA	[23]
	*recA*-R	TTACCAAACATCACGCCTAT	

### Antimicrobial susceptibility

Antimicrobial susceptibility of *H. influenzae* isolates to 10 antibiotics, including ampicillin (AMP), ampicillin–sulbactam (AMS), ciprofloxacin (CIP), levofloxacin (LEV), ceftriaxone (CTR), cefuroxime (ROXH), cefotaxime (TAX), meropenem (MEM), trimethoprim–sulfamethoxazole (SXT), azithromycin (AZT), was determined by Kirby–Bauer disk diffusion test (Oxoid, United Kingdom) on *Haemophilus* test medium (HTM) plates and incubated for 24 h at 37 °C in air with 5% CO_2_. All the isolates were tested for the production of β-lactamase by nitrocephin paper disks.

Isolates that showed susceptibility to ciprofloxacin and had an inhibitory zone diameter of 21–28 mm were screened with nalidixic acid (NAL) disks (30 μg). Minimum inhibitory concentrations (MICs) of ciprofloxacin and levofloxacin were detected by E-test method (AB bioMerieux) in isolates resistant to ciprofloxacin or NAL disks. Susceptibility results were interpreted according to CLSI standards 26th Edition except NAL screening. NAL screening was interpreted according to EUCAST criteria [16]. *H. influenzae* was considered decreased susceptibility to ciprofloxacin when MICs of ciprofloxacin were ≥ 0.125 μg/ml. *H. influenzae* ATCC 29247 was used as a quality control strain for susceptibility testing.

### Sequencing of QRDRs and plasmid-mediated quinolone resistance genes

*Haemophilus influenzae* showed decreased susceptibilities to ciprofloxacin were selected for molecular analysis. The isolates were detected for *gyrA*, *gyrB*, *parC* and *parE* mutations by using primers and methods described previously [25]. The amplification condition for QRDRs was as follows: denaturation at 95 °C for 5 min and 30 cycles of 95 °C for 30 s, annealing at 60 °C for 1 min, and polymerization at 72 °C for 90 s; The samples were then extended at 72 °C for 7 min to fully extend the amplicons. The sequences of the PCR products were compared with known sequences *H. influenzae* Rd KW20 complete genome (L42023) in the GenBank. Plasmid mediated quinolone resistance (PMQR) genes *qnrA*, *qnrB*, *qnrS*, *aac(6′)*-*Ib*-*cr*, *qepA, oqxA*, and *oqxB* were amplified and sequenced as described previously [26] (Table 1).

### Nucleotide accession numbers

The sequences of 11 strains with *gyrA* or *parC* mutations have been annotated and submitted to the GenBank under accession number MG694554, MG694555, MG694556, MG694557, MG694558, MG694559, MG694560, MG694561, MG694562, MG694563 and MG694564.

### Multilocus sequence typing (MLST)

Seven housekeeping genes fragments including *adk*, *atpG*, *frdB*, *fuck*, *mdh*, *pgi*, *recA* were amplified according to the primers in Table 1. PCR products were obtained with the following PCR amplification procedure, initial denaturation at 95 °C for 4 min, followed by 30 cycles of 95 °C for 30 s, 55 °C for 30 s, and 72 °C for 60 s. The samples were then maintained at 72 °C for 10 min, cooled to 4 °C. Allele numbers and STs were assigned by applying the *H. influenzae* MLST website (http://haemophilus.mlst.net/) [27].

### Statistical analysis

Data were analyzed with the Statistical Package for Social Sciences for Windows (SPSSWIN, Chicago, IL, USA, version 19.0). Continuous data, shown as mean ± SD or medians, were compared by Student’s t-test or the Mann–Whitney *U*-test. Categorical variables, summarized as numbers and percentages, were compared by Chi square-test or Fisher’s exact test. Two sided *p*-values less than 0.05 were considered statistically significant.

## Results

### Bacterial isolates

As clinical data of the studied 402 nonduplicate *H. influenzae* strains was concerned, male patients accounted for 63.7% (n = 256), and 130 (32.3%), 110 (27.4%) and 162 (40.3%) patients were ≥ 60 (mean: 79.41 ± 8.77), 15–59 (mean: 50.50 ± 15.56) and < 15 (mean: 50.50 ± 15.56) years old, respectively. *H. influenzae* strains were originated from different anatomical sites: sputum (n = 258, 64.1%), throat swab (n = 64, 15.9%), bronchoscope sputum (n = 48, 11.9%), bronchoalveolar lavage fluid (n = 19, 4.7%), secretion (n = 13, 3.2%). *H. influenzae* isolates were obtained from patients admitted to the respiratory department (n = 141, 35.0%), pediatrics department (n = 163, 40.5%), otolaryngology department (n = 12, 3.0%), thoracic surgery (n = 10, 2.5%), nephrology department (n = 8, 2%), allergy department (n = 7, 1.7%), radiotherapy department (n = 7, 1.7%), others (n = 54, 13.4%).

### Sensitivity of *H. influenzae* isolates

Of the 402 *H. influenzae* isolates, one hundred and sixty-nine (42.0%) possessed β-lactamases and were resistant to ampicillin. Eighty-nine isolates were β-lactamase negative but resistant to ampicillin which were defined as BLNAR (β-lactamase-negative ampicillin-resistant). Forty isolates were totally susceptible to ten antimicrobials according to Kirby–Bauer disk diffusion test, while 259 (64.4%) strains were resistant to sulfamethoxazole, followed with ampicillin (n = 247, 61.4%), cefuroxime (n = 114, 28.4%), azithromycin (n = 70, 17.4%), cefotaxime (n = 45, 11.2%), ceftriaxone (n = 37, 9.2%), meropenem (n = 21, 5.2%). Multidrug resistant strains accounted for 61 (15.2%).

### Ciprofloxacin-nonsusceptible *H. influenzae* isolates

Eleven (2.7%) ciprofloxacin-resistant *H. influenzae* were detected by the Kirby–Bauer disk diffusion test and seven (1.7%) with reduced susceptibilities or resistant to ciprofloxacin (MICs were range from 0.5 to 12 μg/ml) were detected by the E-test method. The MICs of levofloxacin ranged from 0.5 to 3 μg/ml. Among 402 *H. influenzae*, fifteen (3.7%) isolates with ciprofloxacin inhibitory zone diameters of 21–28 mm were screened by the NAL disks, 2 of which were NAL-resistant with inhibitory zone diameters less than 23 mm. The ciprofloxacin MICs of NAL-resistant strains were 0.38 μg/ml and 0.5 μg/ml, respectively. The susceptibility of ciprofloxacin-nonsusceptible *H. influenzae* isolates were shown in Table 3.

### Infection risks analysis

The comparison of ciprofloxacin nonsusceptible to susceptible group according to 17 clinical variables is listed in Table 2, which is including sex, underlying diseases, causes, and medical history during previous 3 months. Among the causes, receiving invasive procedure like sputum suction and bronchoscopy (*p *= 0.011), catching cold or previous contact with someone who had a cold (*p* = 0.019) and fluoroquinolones use during previous 3 months (*p* = 0.003) can increased the risks of ciprofloxacin-nonsusceptible *H. influenzae* infection (Table 2). Moreover, multidrug resistant strains were more common to the ciprofloxacin-nonsusceptible group (*p *= 0.000).

**Table 2:** Clinical characteristics and infection risk factors of *Haemophilus influenzae* with resistance or reduced susceptibility to ciprofloxacin/levofloxacin

Variables^a^	N	Ciprofloxacin^c^		p^d^
		Susceptible	Decreased susceptibility	
Age (years)				
0–14	162 (40.3%)			
5–59	110 (27.4%)			
≥ 60	130 (32.3%)			
Sex				
Male	256 (63.7%)	250 (63.6%)	6 (66.7%)	1.000
Female	146 (36.3%)	143 (36.3%)	3 (33.3%)	1.000
Prevalent seasons (months)				
3–5	138 (34.3%)			
6–8	67 (16.7%)			
9–11	54 (13.4%)			
12–2	143 (35.6%)			
Underlying diseases				
Chronic obstructive pulmonary disease	60 (14.9%)	58 (14.7%)	2 (22.2%)	0.626
Hypertension	70 (17.4%)	68 (17.3%)	2 (22.2%)	1.000
Diabetes	28 (7.0%)	27 (6.9%)	1 (11.1%)	1.000
Chronic kidney disease	33 (8.2%)	33 (8.4%)	0 (0%)	1.000^b^
Cancers	46 (11.4%)	45 (11.5%)	1 (11.1%)	1.000
Interstitial lung Disease	14 (3.5%)	1 3(3.3%)	1 (11.1%)	0.732
Bronchiectasia	27 (6.7%)			
Causes				
Catch cold or be tired	30 (7.5%)	27 (6.9%)	3 (33.3%)	0.019
Invasive procedure	27 (6.7%)	24 (6.1%)	3 (33.3%)	0.011
History of smoking	40 (9.9%)	39 (9.9%)	1 (11.1%)	1.000
Channel of infection				
Community-acquired	372 (92.5%)			
Hospital-acquired	30 (7.5%)			
Hospitalization during previous 3 months	133 (33.1%)	128 (32.6%)	5 (55.6%)	0.147
Fluoroquinolones use during previous 3 months	5 (1.2%)	7 (1.8%)	2 (22.2%)	*0.003*
Antimicrobial use during previous 3 months				
Cephalosporins		144 (36.6%)	4 (44.4%)	0.896
Azithromycin		70 (17.8%)	0 (0%)	0.370^b^
Piperacillin		13 (3.3%)	1 (11.1%)	0.732
Multidrug resistant strains		55 (14.0%)	8 (88.9%)	0.000

^a^ Data are presented as mean ± SD or number of patients (%)

^b^Fisher’s exact test

^c^Numbers of susceptible and decreased susceptibility are 393 and 9, respectively

^d^Statistically significant data are in italics

### Detection of amino acid substitutions in the QRDRs and plasmid-mediated quinolone resistance genes

Seven isolates harbored the amino acid substitution Ser-84-Leu in *gyrA* while four isolates harbored the amino acid substitution Ser-84-ILe in *parC*. Four isolates had both two amino acid substitutions in QRDRs. Three of the isolates with mutations were obtained from young children with 1–3 years of age. The MICs of ciprofloxacin to strains with one or two mutations were of 0.38–1.5 μg/ml, while one strain obtained from an outpatient with MIC of 12 μg/ml did not harbored alterations in QRDRs. No amino acid substitutions were found in *gyrB* and *parE* genes and no plasmid-mediated quinolone resistance genes were found in our investigation (Table 3).

**Table 3:** Clinical characteristics, resistance and molecular typing of CIP resistant or susceptibility-decreased *H. influenzae* strains

No.	Age	Sex	Date	Department and infection	Causes and underlying diseases	Disk diffusion			E-test		Resistance	During previous 3 months	ST	QRDRs	
						Ci	L	Na	Ci	L		Hospitalization and antimicrobial		*gyrA*	*parC*
38	3	M	17.2.14	Respiratory RI, A	Contact with patient; rhinallergosis	28	25	6	0.38	0.38	AmCfCtST	N; Cf	12	Ser-84-Leu	–
254	70	M	16.5.16	Respiration (4), A	/; interstitial lung disease arthritis deformans	21	19	6	0.5	0.5	AmAsCfS	N; N	836	–	–
179	61	F	16.4.3	Gastrointestinal surgery, B	Radical mastectomy; mammary cancer cholecystolithiasis hypertension	19	21	–	1.5	0.75	CfST	Y; Cf	408	Ser-84-Leu	Ser-84-Ile
187	2	F	16.4.7	Pediatrics, A	Bronchoscopy; acleistocardia	20	6	–	1	0.5	CfS	Y (3); Cf	408	Ser-84-Leu	Ser-84-Ile
197	1	M	16.4.8	Pediatrics, A	Bronchoscopy; tracheostenosis, acleistocardia family history of rhinallergosis	19	23	–	1	0.75	CfS	Y (4); unspecified fluoroquinolone, Cf	408	Ser-84-Leu	Ser-84-Ile
216	62	M	16.4.21	Respiration (2), A	Catch cold, COPD, hypertension	20	21	–	0.5	0.5	AmAsAzS	Y; fluoroquinolone enoxacin, P	422	Ser-84-Leu	--
237	87	F	16.5.3	Respiration (3), A	Mechanical ventilation and catch cold; smoking COPD chronic corpulmonale	19	21	–	0.75	0.5	AmAsAzCfCtST	Y; N	422	Ser-84-Leu	-
291	61	M	16.7.12	Respiration (3), A	/; emphysema	20	6	–	0.75	0.5	AmAsAzCfST	N; N	408	Ser-84-Leu	Ser-84-Ile
387	56	M	16.10.10	Outpatient, A	Catch cold; /	6	18	–	> 12	3	M	/; N	1791	–	–

Figures in brackets after department means different zones of the department. Figures in brackets of column Hospitalization means the frequency of hospitalization during previous 3 months

*M* male, *F* female, / not mentioned, *Y* yes, *N* no, *A* community-acquired, *B* hospital-acquired, *Am* ampicillin, *As* ampicillin–sulbactam, *Az* azithromycin, *Ci* ciprofloxacin, *Cf* cefuroxime, *Ct* ceftriaxone, *M* meropenem, *Na* nalidixic acid, *L* levofloxacin, *P* piperacillin, *S* trimethoprim–sulfamethoxazole, *T* cefotaxime, *QRDR* quinolone resistance determining regions, *ST* sequence

### Multilocus sequence typing (MLST)

Nine ciprofloxacin-nonsusceptible isolates were resolved into 5 different sequence types (ST), which displayed high genetic variability. Four STs were previously described in the MLST database and one (ST1791) was a new ST that had not been reported previously. ST408 was the most common ST in our study. However, according to the epidemiological data such as the isolation date and departments, no clonal spread of FRHI was found in our study.

## Discussion

This study has provided data of ciprofloxacin-resistant *H. influenzae* incidence in Guangzhou, China. The incidence in our study was closer to the report from Hong Kong [6], which stayed at a very low resistant rate, in contrary to Taiwan’s [4, 8]. According to our investigation, the use of fluoroquinolone was not so common in adults, which may explain the low incidence of ciprofloxacin-resistant *H. influenzae* in Guangzhou. Although most of the *H. influenzae* were community-acquired, invasive procedure like sputum suction and bronchoscopy may also increase the infection risks of ciprofloxacin-resistant *H. influenzae* which indicated that nosocomial environment colonization is an important route of ciprofloxacin-resistant *H. influenzae* infection. In addition, medication history of fluoroquinolone during previous 3 months is closely related to ciprofloxacin-resistant *H. influenzae* infection. Patients who are reinfected with *H. influenzae* should be monitored of fluoroquinolone susceptibility for the isolates may have obtained resistance under antibiotic selective pressure. It is known that fluoroquinolone is not approved for children in most regions of the world now, and children infected with ciprofloxacin-resistant *H. influenzae* in our study were all community-acquired, so we inferred that the acquisition of such *H. influenzae* in children may be attributed to disseminating from community environment and consuming products from food animal previously fed antimicrobial agents of fluoroquinolone. In addition, *H. influenzae* carriage in children should rise our concern in preventing the spread of resistant *H. influenzae*, especially in public areas like daycares [6].

Although ciprofloxacin-resistant *H. influenzae* remains rare in Guangzhou, nine ciprofloxacin-nonsusceptible. *H. influenzae* isolates were detected in our study (2.2%, including two NAL-resistant strains), which may develop into resistance in a stepwise manner according to the molecular mechanism in QRDRs. Seven of the isolates had developed amino acid substitution in QRDRs, with ciprofloxacin MICs of 0.38 to1.5 μg/ml. *H. influenzae* in our investigation with ciprofloxacin MICs of 0.38–0.75 μg/ml had one amino acid alteration in *gyrA*, and isolates with ciprofloxacin MICs of 0.75–1.5 μg/ml had two amino acid alteration in *gyrA* and *parC*. According to the previous report, isolates with ciprofloxacin MICs of 0.12–0.5 μg/ml had at least one amino acid alteration in a position of the fluoroquinolone targets involved in resistance. It was revealed that the degree of fluoroquinolone-resistance is related to the number of the mutations in QRDRs [16]. Strains presenting one or two mutations in *gyrA* and *parC* have low-level resistance to fluoroquinolone, while those with three or more mutations show high-level resistance [28]. In general, our finding was consistent with the report [16]. However, there were two isolates with ciprofloxacin MICs of 0.5 μg/ml and 12 μg/ml respectively didn’t harboring any mutations in QRDRs and plasmid-mediated quinolone resistance genes. We inferred that some other mechanisms may participated in fluoroquinolone-resistance of *H. influenzae*.

Amino acid changes in *gyrA* residue (Ser84) and/or *parC* residue (Ser84) were detected in our study while no substitutions occurred in *gyrB* and *parE*. Ser84Leu in *gyrA* and Ser-84-Ile in *parC* were globally reported according to the current literatures, including Spain, Japan, Taiwan [4, 14, 16, 25, 29]. The similar mutation patterns in QRDRs around different areas indicated that Ser-84-Leu in *gyrA* and Ser-84-Ile in *parC* were one of the most common mutations in QRDRs. Comparing with the mutation profiles in QRDRs from Guangzhou and Taiwan, the latter one had more diverse mutation profiles than Guangzhou [4]. In Taiwan, emergence of fluoroquinolone-resistant isolates may be the results of several clones spreading in the same region. However, according to our investigation, no clone transmitted hospital infection was found which indicated that fluoroquinolone-nonsusceptible *H. influenzae* may occur sporadically in Guangzhou presently. In addition, our investigation reported a relative high genetic diversity among fluoroquinolone-resistant *H. influenzae* isolates. None of the STs published in Taiwan were found in our study, but ST422 and ST408 involved in our study were previously reported in Sichuan, China, where also discovered a series of diverse and novel sequence types as Taiwan [4, 30]. The predominant epidemiological STs in China like ST486 and 480 were not found in our study. The heterogeneous characteristics of the *H. influenzae* strains may be the reason that no outbreak or epidemics occurred in Guangzhou. Moreover, high heterogeneity of *H. influenzae* strains by MLST analysis revealed that the *H. influenzae* strains were regionally varied and evolved independently. One strain was confirmed as novel sequence type (ST1719) in our study, which showed high-level resistance of ciprofloxacin while no mutations in QRDRs was found.

There were two NAL-resistant strains harboring amino acid substitution in QRDRs in our study and both of them were ciprofloxacin-susceptible according to CLSI criteria. It has been argued that, the application of current CLSI criteria may underrecognize the low-level ciprofloxacin-resistant *H. influenzae* with first step mutations in QRDRs [6, 16, 17]. As a screening test for determination of the susceptibilities of gram-negative bacteria to fluoroquinolone, NAL has drawn special attention [6, 16], it can separate strains with reduced quinolone susceptibilities as a result of the acquisition of mutations in the QRDRs. However, NAL screening was not applied routinely in determination of the susceptibilities of *H. influenzae* in clinics which may underrecognize a proportion of strains with decreased fluoroquinolone susceptibilities. In addition, no acceptable criteria for NAL screening was established by CLSI in the current. In order to detect fluoroquinolone non-susceptible strains with first step mutation in QRDRs, screening with NAL as an indicator of resistance was proposed by the EUCAST guidelines. First failure case of oral levofloxacin treatment of community-acquired pneumonia caused by *H. influenzae* had been reported in 2003 [14]. After that, reports on fluoroquinolone treatment failure were occurred globally. An reports from New York declared that persistent stimulation of low concentration fluoroquinolone in fluoroquinolone-susceptible *H. influenzae* may increase the fluoroquinolone resistance of *H. influenzae* isolates which offered experiment evidence for the declaration of avoiding fluoroquinolone treatment in *H. influenzae* close to breakpoints. And they pointed out that the key to preventing fluoroquinolone resistance in *H. influenzae* may be to block enrichment of the first *gyrA* mutation by strictly avoiding low dose fluoroquinolones use [28]. In addition, medication history of fluoroquinolone is related to fluoroquinolone non-susceptible *H. influenzae* infection according to our investigation, so we inferred that a proportion of strains harboring the first step mutation in QRDRs might obtain fluoroquinolone resistance under fluoroquinolone selection pressure. High-level resistance might be induced by repeated treatment with fluoroquinolone in patients with chronic lung diseases like COPD. Treatment failure reminded us that we should pay attention to those *H. influenzae* with resistance or decreased susceptibilities to fluoroquinolone, and should facilitate the laboratory detection of strains with decreased susceptibilities to fluoroquinolone.

In conclusion, as the first study of FRHI in mainland China, reduced susceptibilities to fluoroquinolones had been obtained, despite low incidence of FRHI isolates. Invasive procedure and medication history of fluoroquinolone during previous 3 months can increase the infection risks of FRHI. High genetic diversity in FRHI strains has strongly suggested their sporadic emergence and spread in Guangzhou of Southern China, and amino acid substitutions in QRDRs of strains with decreased susceptibilities to fluoroquinolone have also presented evidence on the initial evolution of fluoroquinolone resistance by mutation in *gyrA*, indicating the potential treatment failure or increase of fluoroquinolone resistance under continuous fluoroquinolone therapy. In addition, this study has for the first time reported the mutation of *gyrA* and *parC* in mainland China and the new ST1719 worldwide, which may further raise clinical concerns regarding the therapeutic efficacy of fluoroquinolone treatment and aid in further investigation on FRHI.

## Abbreviations

FRHI: fluoroquinolone-resistant *Haemophilus influenzae*
QRDR: quinolone resistance-determining regions
CAP: community-acquired pneumonia
AOM: acute otitis media
FQs: fluoroquinolones
ST: sequence types
AMP: ampicillin
AMS: ampicillin–sulbactam
CIP: ciprofloxacin
LEV: levofloxacin
CTR: ceftriaxone
ROXH: cefuroxime
TAX: cefotaxime
MEM: meropenem
SXT: trimethoprim–sulfamethoxazole
AZT: azithromycin
HTM: *Haemophilus* test medium
NAL: nalidixic acid
MICs: minimum inhibitory concentrations
PMQR: plasmid mediated quinolone resistance
